# Evolution of microbes and viruses: a paradigm shift in evolutionary biology?

**DOI:** 10.3389/fcimb.2012.00119

**Published:** 2012-09-13

**Authors:** Eugene V. Koonin, Yuri I. Wolf

**Affiliations:** National Center for Biotechnology Information, National Library of Medicine, National Institutes of HealthBethesda, MD, USA

**Keywords:** Darwin, modern synthesis, comparative genomics, tree of life, horizontal gene transfer

## Abstract

When Charles Darwin formulated the central principles of evolutionary biology in the *Origin of Species* in 1859 and the architects of the Modern Synthesis integrated these principles with population genetics almost a century later, the principal if not the sole objects of evolutionary biology were multicellular eukaryotes, primarily animals and plants. Before the advent of efficient gene sequencing, all attempts to extend evolutionary studies to bacteria have been futile. Sequencing of the rRNA genes in thousands of microbes allowed the construction of the three- domain “ribosomal Tree of Life” that was widely thought to have resolved the evolutionary relationships between the cellular life forms. However, subsequent massive sequencing of numerous, complete microbial genomes revealed novel evolutionary phenomena, the most fundamental of these being: (1) pervasive horizontal gene transfer (HGT), in large part mediated by viruses and plasmids, that shapes the genomes of archaea and bacteria and call for a radical revision (if not abandonment) of the Tree of Life concept, (2) Lamarckian-type inheritance that appears to be critical for antivirus defense and other forms of adaptation in prokaryotes, and (3) evolution of evolvability, i.e., dedicated mechanisms for evolution such as vehicles for HGT and stress-induced mutagenesis systems. In the non-cellular part of the microbial world, phylogenomics and metagenomics of viruses and related selfish genetic elements revealed enormous genetic and molecular diversity and extremely high abundance of viruses that come across as the dominant biological entities on earth. Furthermore, the perennial arms race between viruses and their hosts is one of the defining factors of evolution. Thus, microbial phylogenomics adds new dimensions to the fundamental picture of evolution even as the principle of descent with modification discovered by Darwin and the laws of population genetics remain at the core of evolutionary biology.

## Introduction

Charles Darwin's *On the Origin of Species* that appeared in London in 1859 (Darwin, 1859) was the first plausible, detailed account of biological evolution, after the simultaneous and independent brief outlines by Darwin and Alfred Russell Wallace that were published the previous year (Darwin, 1858; Wallace, 1858). Darwin did not discover evolution and did not even offer the first coherent description of evolution: exactly 50 years before the appearance of the *Origin*, the French botanist and zoologist Jean-Baptiste Lamarck published his magnum opus *Philosophie Zoologique* (Lamarck, 1809) in which he outlined his vision of the history of life in considerable detail. However, the cornerstone of Lamarck's worldview was the purported intrinsic drive of evolving organisms toward “perfection,” a patently non-scientific, irrational idea. Moreover, Lamarck's view of the role of evolution in the history of life was severely limited: he did not postulate deep common ancestry of life forms but rather believed in multiple acts of creation, perhaps a separate act for each species. Prescient ideas on evolutionary changes of organisms actually have been developed centuries before Lamarck and Darwin, most notably by the great Roman thinker Titus Lucretius Carus (2011).

However, the fact remains that it was Darwin's first evolutionary synthesis that had launched the field of evolutionary biology in a sense close to the modern one and had remained central to biological thinking over the last 150 years inasmuch as “nothing in biology makes sense except in the light of evolution” (Dobzhansky, 1973). Darwin's concept lacked the essential foundation in genetics for the obvious reason that mechanisms of heredity were unknown in his day. Hence Darwin's deep concern over the so-called Jenkin nightmare, the objection to Darwin's concept according to which beneficial changes would be “diluted” after several generations in the progeny of organisms in which they occurred. The genetic basis of evolution was established after the rediscovery of Mendel's laws, with the development of population genetics in the first third of the twentieth century, primarily, through the pioneering work of Fisher, Wright, and Haldane (Fisher, 1930; Haldane, 1932). The new, advanced understanding of evolution, informed by theoretical and experimental work in genetics, was consolidated in the Modern Synthesis of evolutionary biology, usually, associated with the names of Dobzhansky, Julius Huxley, Mayr, and Simpson (Dobzhansky, 1937; Simpson, 1944). Apparently, the Modern Synthesis reached its mature form during the 1959 centennial celebration for the *Origin* in Chicago (Tax and Callender, 1960; Browne, 2008).

Now, 50 years after the consolidation of the Modern Synthesis, evolutionary biology undoubtedly faces a new major challenge and, at the same time, the prospect of a new conceptual breakthrough (Rose and Oakley, 2007). If the Modern Synthesis can be succinctly described as Darwinism in the Light of Genetics (often referred to as neodarwinism), then, the new stage is Evolutionary Biology in the Light of Genomics and Microbiology. The combination of genomics and microbiology is indeed critical in the advent of this new age of evolutionary biology (Koonin and Wolf, 2008; Koonin, 2009a; Woese and Goldenfeld, 2009). Lamarck and Darwin (let alone Lucretius) were plainly unaware of the existence of genomes and microbes. The architects of the Modern Synthesis certainly knew about genomes and microbes “in principle” but, in the former case, did not know enough to incorporate information on genomes beyond the (important but limited) level of formal genetics, and in the latter case, did not realize the importance of microbes for understanding evolution at all.

In this article, we attempt to outline the key changes to the basic tenets of evolutionary biology brought about primarily by comparative and functional microbial genomics and argue that, in many respects, the genomic stage could be a more radical departure from the Modern Synthesis than the latter was from classic Darwinian concepts.

## From the tree of life to the web of gene trees

The famous sole illustration of the Origin of Species shows a Tree of Life (or more precisely, a series of trees presumably depicting the evolution of different divisions of organisms). Obviously, Darwin was not the first to use a tree to depict history. Before him, trees had been employed for many centuries to capture human genealogy, e.g., that of the Old Testament patriarchs as well as later monarchs. Darwin, however, was the first to make the crucial conceptual step by boldly proposing that the entire history of life could (at least in principle) be accurately represented by a tree growing from a single root. Darwin's tree was a sheer scheme, without any attempt to assign real life forms to the branches but in just a few years Ernst Haeckel populated the tree by a huge variety of organisms, almost exclusively animals (Haeckel, 1997). Haeckel inferred the relationships between organisms reflected in the topology of his tree primarily on the data of comparative anatomy that was already advanced in his day. Over the next century, there was considerable progress in this field leading to improved resolution of the tree but qualitatively the situation has not changed. Phylogeny largely served as a tool for systematics, and the architects of the Modern Synthesis were much more interested in mechanisms of microevolution and speciation than in the course of macroevolution that is supposedly reflected in the Tree of Life. Although by mid-twentieth century microbiologists had realized full well that microbes possess genomes and can mutate, and accordingly, should evolve, in principle, similarly to animals and plants, all attempts to infer microbial evolution from morphological and physiological characters had been unqualified failures (Stanier and Van Niel, 1962).

**Figure 1:**
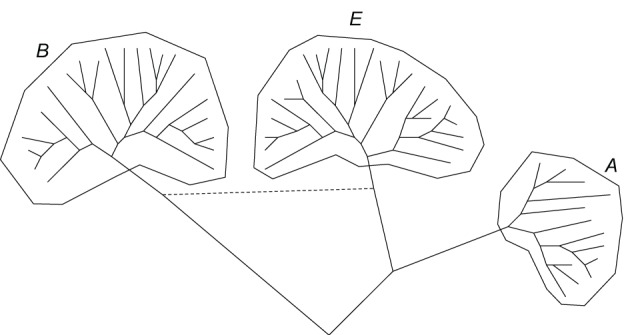
**The three-domain tree of life: a generalized schematic, A, Archaea, B, bacteria, E, Eukaryota**.

The fortunes of phylogeny and microbial evolution changed abruptly in the late 1970s when Carl Woese and colleagues realized that the nucleotide sequence of a universally conserved molecule, 16S rRNA, could be used to infer a universal phylogenetic tree (rather incredibly, from today's vantage point, Woese's original seminal work employed oligonucleotide maps of 16S RNA rather than sequences; however, the actual sequences became readily available shortly, and the main conclusions of the early studies stood) (Woese, 1987). Comparison of 16S RNA sequences had swiftly led to the discovery of a distinct domain of life, the Archaea, and its distinct phylogenetic affinity with the eukaryotes (Woese and Fox, 1977; Woese et al., 1990; Woese, 2004). Over the following few years, major phyla of Bacteria, Archaea and unicellular eukaryotes have been established (Woese, 1987), and the famous tripartite tree (Figure 1) emerged as the paradigm of the history of cellular life on earth which it more or less remains to this day (Woese et al., 1990; Pace, 1997, 2006, 2009). This was a veritable triumph of molecular phylogenetics and a dramatic departure from Haeckel's Tree of Life. In Haeckel's tree, Protista (unicellular eukaryotes) and Monera (bacteria) occupied unspecified positions near the root. For all purposes, these measly, tiny creatures were not considered important in the big picture of evolution. The tripartite tree of Woese and colleagues was a complete change of perspective. Now, two of the three domains of life were represented by prokaryotes (former Monera), and within the eukaryote domain, the majority of the phyla were represented by unicellular organisms (former Protista). The life forms formerly considered “important,” i.e., the complex multicellular organisms (animals and plants), represent only two among the numerous branches of eukaryotes. There is no denying the fact that the true biodiversity on this planet is the diversity of unicellular microbes.

In the 1980s, when the paradigmatic status of the three-domain Tree of Life was established, there was little concern over the fact that technically this tree represented the history of only one gene, even if a universally present and highly conserved one. The 16S RNA was unanimously considered a suitable reference gene to represent the evolution of the respective organisms. Other universal genes, such as 18S RNA ribosomal proteins or RNA polymerase subunits, were thought to be important only to the extent their inclusion could improve the resolution of phylogenetic trees.

Even long before the advent of the genomic era, microbiologists realized that bacteria had the capacity to exchange genetic information via horizontal gene transfer (HGT), in some cases, producing outcomes of major importance, such as antibiotic resistance (Syvanen and Kado, 2002). Multiple molecular mechanisms of HGT have been described including plasmid exchange, transduction (HGT mediated by bacteriophages), and transformation (Bushman, 2001) [indeed, the phenomenon of transformation was employed by Avery and colleagues to demonstrate the genetic function of DNA in 1944 (Avery et al., 1944a)]. However, despite these discoveries, HGT was generally viewed as a minor phenomenon that was important only under special circumstances and, in any case, did not in any manner jeopardize the Tree of Life that could be reconstructed by phylogenetic analysis of rRNA and other conserved genes.

This comfortable belief was abruptly shattered when the early findings of comparative genomics of bacteria and archaea in the late 1990s have indicated that, at least in some prokaryotic genomes, a substantial fraction of genes were acquired via demonstrable HGT, sometimes across log evolutionary distances. The pathogenicity islands and similar symbiosis islands that comprise over 30% of the genome in many pathogenic and symbiotic bacteria and obviously travel between bacteria via HGT are the prime case in point (Hacker and Kaper, 2000; Perna et al., 2001). Perhaps, more strikingly, comparative analysis of the genomes of hyperthermophilic bacteria and archaea has suggested that in shared habitats even HGT between the two domains of prokaryotes, Archaea and bacteria, can be extensive, with up to 20% of the genes of bacterial hyperthermophiles showing archaeal affinity (Aravind et al., 1998; Nelson et al., 1999; Koonin et al., 2001). Subsequent phylogenomic studies (that is analysis of phylogenies of multiple genes from numerous genomes) have led to a shocking realization: in prokaryotes at least, there seem not to exist two genes with the exact same evolutionary history (Koonin et al., 2001; Gogarten and Townsend, 2005; Gribaldo and Brochier, 2009; Zhaxybayeva, 2009; Boto, 2010; Andam and Gogarten, 2011; Zhaxybayeva and Doolittle, 2011). Apparently, this is so because all genes have experienced HGT at some stage (s) of their evolution. Although some genes, in particular those that encode components of the translation system, show substantial congruency (but not actual identity) between each other and with the standard rRNA tree, the number of such congruent trees is small. In a memorable phrase of Bill Martin and Tal Dagan, the ribosomal tree of a life is at best “a tree of one percent” (of all genes in microbial genomes) (Dagan and Martin, 2006).

**Figure 2:**
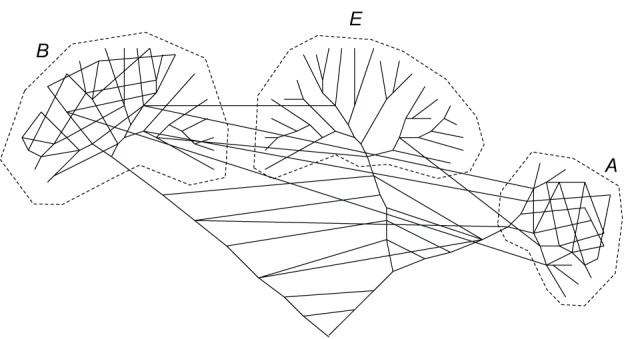
**A network representation of the evolutionary process.** The network still includes some tree components such that the three domains of cellular life remains distinct but there is also an extensive horizontal component of genetic information flow that in particular dominates the earliest stages of evolution (Koonin and Wolf, 2008).

Thus, “evolution of prokaryotes and the Tree of Life are two different things” (Bapteste et al., 2009; Martin, 2011). Then, the question arises: is there any substantial tree component in evolution at all and accordingly does it make any sense to speak of HGT? Indeed, horizontal transfer can be defined as such only against some standard of vertical evolution (Bapteste et al., 2005; Doolittle and Bapteste, 2007; Bapteste and Boucher, 2009). As Martin and Dagan wryly notice, if a model (in this case, the Tree of Life model) adequately describes 1% of the data, it might be advisable to abandon it and search for a better one (Dagan and Martin, 2006). Such an alternative indeed has been proposed in the form of a dynamic network of microbial evolution in which the nodes are bacterial and archaeal genomes, and the edges are the fluxes of genetic information between the genomes (Kunin et al., 2005; Dagan and Martin, 2009; Dagan, 2011; Kloesges et al., 2011). In the extreme, such a network has no vertical, tree-like component whereas the weights of the edges differ depending on the intensity of the gene exchange (Figure 2). Moreover, it has been persuasively argued that “tree thinking in biology” might be a sheer myth, however deeply entrenched in the textbooks and the minds of biologists (Bapteste et al., 2005; Doolittle and Bapteste, 2007; Bapteste and Boucher, 2009). Indeed, there is potential for tree-like patterns to emerge from relationships that have nothing to do with common descent as exemplified by Doolittle and Bapteste by the distribution of human names across the departments of France (Doolittle and Bapteste, 2007).

One could argue, however, that the tree pattern is not at all illusory but, on the contrary, is intrinsic and central to the entire process of biological evolution. The relevance and generality of this pattern plainly follows from the fundamental character of the replication process that underlies the evolution of life (Koonin and Wolf, 2009b). Successive generations of replicating genomes (and accordingly, dividing cells) follows an inherently binary branching pattern that, over generation naturally yields a tree. The tree pattern is predicated on a low rate of intragenic recombination which is indeed the case for all evolutionary distances large enough to prevent homologous recombination. Accordingly, evolutionary history of individual genes can be adequately represented by trees (the practical problems of accurate phylogeny reconstruction notwithstanding).

A natural, key question to ask then is: are the topologies of the trees for individual genes substantially congruent? In other words, is it possible to identify a statistically significant central trend in the vast “forest” of gene trees? Statistical analysis of thousands of phylogenetic trees for diverse genes of prokaryotes (in fact, all genes with sufficient degree of conservation to obtain a reliable tree topology) has shown that a highly significant central trend is indeed detectable in the phylogenetic forest (Puigbo et al., 2009, 2012; Koonin et al., 2011). Moreover, the consensus topology of the supertree of the (nearly) universal genes (the notorious 1%) turned out to be the best approximation of that central trend. Thus, although any phylogenetic tree of a central, conserved component of the cellular information-processing machinery (such as rRNA or the set of universal ribosomal proteins) represents only a minority of the phylogenetic signal across the phylogenetic forest (see details below) and so by no account can be considered an all-encompassing “Tree of Life,” neither is such a phylogeny an arbitrary and irrelevant “tree of 1%.” On the contrary, these trees represent a central evolutionary trend and reflect a “statistical tree of life” (O'Malley and Koonin, 2011).

## The dynamic gene universe

For decades microbiologists knew that bacteria sometimes exchange genes (Low and Porter, 1978; Arber, 1979; Campbell, 1981; Syvanen, 1985, 1994). Moreover, the phenomena of transformation, acquisition of new traits via import of DNA from the environment and integration of the imported molecules into the bacterial genome, and transduction, transfer of genetic markers by bacteriophages, have been studied in considerable detail. In fact, transformation was the basis of the seminal 1944 experiments of Avery and colleagues which demonstrated that the genetic material of bacteria consisted of DNA (Avery et al., 1944b). In addition, microbiologists realized that such HGT could exert well-defined, major biological effects such as conferring pathogenicity (as in Avery's experiments) or antibiotic resistance on the recipients of horizontally transferred genes. However, all this knowledge notwithstanding, in the pregenomic era, HGT was considered a highly specialized genetic pathway rather than the mainstream of microbial evolution.

Comparative genomics brought the shocking realization that bacterial and archaeal genomes were literally shaped by HGT. This was clearly demonstrated by early analyses of the genomes of bacterial hyperthermophiles that were shown contain about 20% of genes of obvious archaeal origin (Aravind et al., 1998; Nelson et al., 1999; Koonin et al., 2001); conversely, genomes of mesophilic Archaea, such as *Methanosarcina*, encompass roughly the same proportion of genes clearly derived from bacteria (Deppenmeier et al., 2002; Galagan et al., 2002). These are striking examples of extensive gene exchange between the most distant prokaryotes that is stimulated by cohabitation. Not unexpectedly, the extent of gene exchange is far greater between more closely related organisms, even if often more difficult to detect (Abby et al., 2012). Nevertheless, phylogenomic analysis of a variety of bacteria and archaea clearly reveals their mosaic origins: different genes affiliate with homologs from different organisms (Koonin et al., 2001; Sicheritz-Ponten and Andersson, 2001; Koonin, 2003; Esser et al., 2007; Koonin and Wolf, 2008; Kloesges et al., 2011). These findings have been encapsulated in the concept of the Rhizome of Life under which the history of any given genome can be represented as a rhizome, with diverse sources and evolutionary histories for different genes (Raoult, 2010; Merhej et al., 2011). Recent, detailed studies indicate that at least in tight microbial communities, such as for instance the human gut microbiota, gene exchange is constant and rampant (Smillie et al., 2011).

In the face of the increasingly apparent genomic promiscuity, one cannot help asking whether “horizontal gene transfer” is a viable concept at all: indeed, for any extended span of evolution, HGT will be identifiable if and only if there is some objectively definable “vertical” standard to compare against. Otherwise, all genetic exchanges would be equal, and the only adequate depiction of evolution would be an undirected network graph. Thus, the validity of the tree representation of evolution and the very existence of HGT are inextricably linked. The results of exhaustive comparison of the individual gene trees in the “phylogenetic forest” discussed in the preceding section reveal the existence of substantial coherence of phylogenetic tree topologies, especially among highly conserved, (nearly) ubiquitous genes that encode components of the translation system (Puigbo et al., 2009). There are many exceptions to this generalization including extensive HGT of genes coding for aminoacyl-tRNA synthetases (Wolf et al., 1999; Woese et al., 2000) and even multiple cases of HGT of genes encoding ribosomal proteins (Brochier et al., 2000; Makarova et al., 2001; Yutin et al., 2012). Nevertheless, these genes appear to comprise a single, co-evolving ensemble, in at least general agreement with the so-called complexity hypothesis (Jain et al., 1999; Wellner et al., 2007; Abby et al., 2012). Under the complexity hypothesis, HGT of genes encoding subunits of macromolecular complexes is largely suppressed because of the deleterious effect caused by disruption of interactions refined by a long time of co-evolution. Indeed, a recent analysis has shown that it is the involvement in complex formation that shows a strong negative correlation with the rate of HGT, rather than any specific biological function (Cohen et al., 2011). Thus, genes encoding many translation system components probably coevolve and accordingly are rarely horizontally transferred because they are preferentially involved in large complexes (above all, the ribosome itself) rather than owing to their special biological importance or any other peculiarities of their biological function. Other genes show a much weaker but also significant phylogenetic coherence with the nearly universal genes for translation system components, perhaps also reflecting the involvement in complex formation.

**Figure 3:**
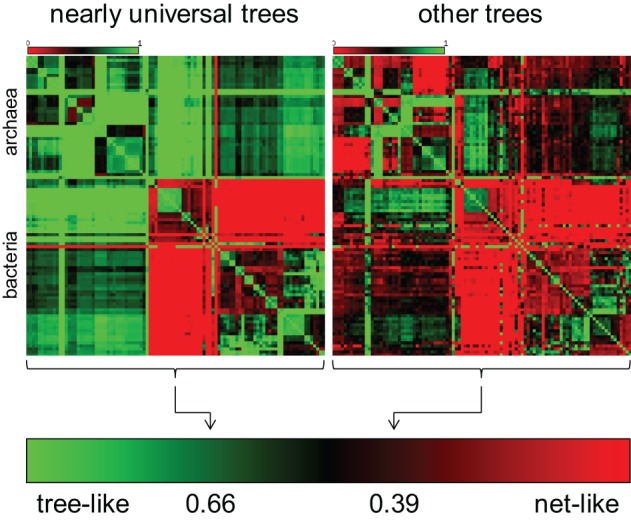
**Tree-like (vertical) and web-like (horizontal) contributions in the evolution of nearly universal genes and the entire phylogenetic forest.** The two heat maps schematically depict comparison of bacterial and archaeal genomes as described previously (Puigbo et al., 2010).

The same series of phylogenomic studies that demonstrated the validity of the statistical tree of life quantified the contributions of tree-like (vertical) and web-like (horizontal) gene transmission to the relationships between bacterial and archaeal genomes (Puigbo et al., 2010, 2012). The results came out remarkably different for the ~100 nearly universal trees and the rest of the trees in the phylogenetic forest. The evolution of the nearly universal trees is dominated by the tree-like trend which contributes approximately 2/3 of the evolutionary information whereas in the rest of the forest, the ratio is the opposite, with about 2/3 of the signal coming from horizontal gene exchange (Figure 3).

The extensive HGT that permeates the prokaryote world is the source of gene gain by bacterial and archaeal genomes. Perhaps, the best characterized case of massive gene gain is the emergence of pathogenic bacterial strains that often evolve by acquiring the so-called pathogenicity islands that sometimes comprise over 30% of the pathogen's genome as first revealed by the comparison of the genomes of laboratory and wild strains of *E. coli* (Perna et al., 2001; Zhang et al., 2007; Eppinger et al., 2011). The opposite trend, gene loss, is at least as prominent as gene gain via HGT (Snel et al., 2002; Mirkin et al., 2003). A prime example is evolution of intracellular parasites and symbionts, for example, *Buchnera*, a close relative of *E. coli* that lost about 90% of the ancestral genes (Perez-Brocal et al., 2006); several other intracellular bacterial parasites and symbionts show even more drastic genome reduction (Klasson and Andersson, 2004; Perez-Brocal et al., 2006; McCutcheon and Moran, 2012).

**Figure 4:**
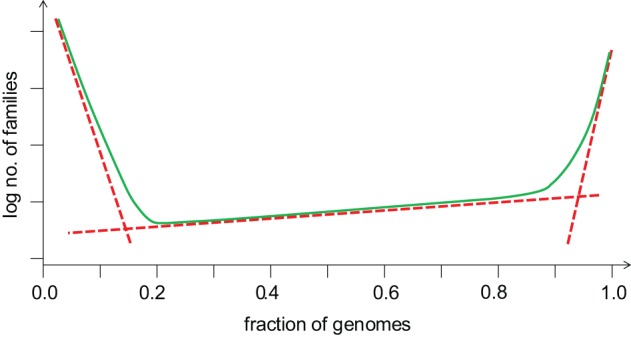
**The universal distribution of gene commonality in the microbial genomic universe: a generalized schematic.** The three broken lines represent three exponential functions that fit the core (on the right), the shell (in the middle) and the cloud (on the left) of prokaryotic genes (O'Malley and Koonin, 2011).

**Figure 5:**
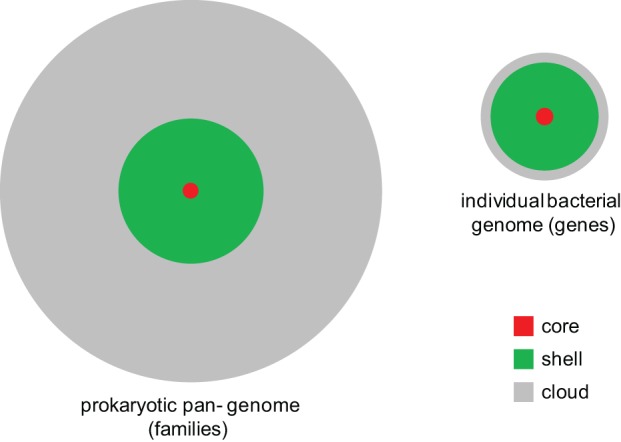
**The core, shell, and cloud of microbial genes.** A generalized schematic showing the approximate contributions of the core, shell, and cloud to the pangenomes of prokaryotes and individual genomes.

The balance between gene gain and gene loss translates into a distinct shape of the distribution of gene occurrence in prokaryote pangenomes at all levels, from closely related bacteria (e.g., those of Enterobacteria) to the entirety of sequenced bacterial and archaeal genomes (Koonin and Wolf, 2008; O'Malley and Koonin, 2011). This universal distribution has an asymmetric U-shape and can be approximated by three exponential functions (Figure 4). The first of these corresponds to a small, highly conserved core (the nearly universal genes discussed above); the second exponent describes the much larger “shell” of genes with limited conservation; and the third one delineates the vast “cloud” of rare, poorly conserved genes. Thus, the gene universe is dominated by rare, sparsely distributed genes most of which are not covered by the limited available sampling of genomes and still remain to be discovered although in each particular genome the moderately conserved “shell” genes comprise the majority (Figure 5). The dynamic, fluid character of the prokaryote genomes yields a distinct, fractal-like structure of the gene universe (O'Malley and Koonin, 2011).

## Are there species in prokaryotes?

The title of Darwin's seminal book “The Origin of Species” is deeply steeped in traditions of eighteenth and nineteenth century biology that tended to view animal and plant species as key units of biological organization. Darwin himself actually saw species more as an arbitrary category in the continuum of varying life forms than a fundamental unit of life. In the twentieth century the species concept received its biological interpretation, primarily in the work of Ernst Mayr who famously defined a species as a system of panmictic populations that are genetically isolated from other such systems (Mayr, 1944). This concept indeed captures a key feature of the biology of organisms with regular, obligatory sexual reproduction such as, above all, animals and to a lesser extent plants.

Most of the prokaryotes do not engage in regular sex but instead exchange genes via HGT with diverse other microbes that they happen to cohabitate with. In general, in the prokaryote world, there are indeed no discrete, genetically isolated systems of panmictic populations but rather complex webs of gene exchange (Dagan et al., 2008; Koonin and Wolf, 2008). Thus, the very notion of species as a distinct biological category does not apply even though traditionally bacteria and archaea are still denoted by Linnaean species names (e.g., *Escherichia coli* or *Haloferax volcanii*) (Konstantinidis et al., 2006; Cohan and Perry, 2007; Doolittle and Zhaxybayeva, 2009; Fraser et al., 2009). However, the modes of evolution substantially differ across the diversity of prokaryotes, spanning the entire continuum from fully sexual to fully clonal populations (Smith et al., 1993; Doolittle and Zhaxybayeva, 2009). Some bacteria, especially parasites such as for example *Neisseria gonorrhoeae*, have been shown to form largely isolated communities that engage in regular conjugation, the bacterial equivalent of sex, resulting in extensive homologous recombination. For these distinct organisms but not for the majority of bacteria and archaea, Mayr's biological definition of species might be a relevant concept.

The irrelevance of the (traditional) species concept for most prokaryotes by no means implies non-existence of structure in the genome space. Indeed, bacteria and archaea that share common origin in phylogenetic trees of marker genes, such as rRNA, typically also possess similar gene content. The “genome-trees” constructed on the basis of the (dis)similarity of gene content are generally congruent with phylogenetic trees of highly conserved marker genes although interesting deviations that reflect similarities in life style and/or extensive gene exchange have been detected as well (Snel et al., 1999, 2005; Wolf et al., 2002).

Thus, although the bacterial and archaeal “species” are not species in the regular sense, they are “galaxies” in the gene universe that form distinct, hierarchical clusters. Interestingly, it has been shown that, among the processes that lead to the divergence of gene content between evolving lineages of prokaryotes, gene loss appears to occur stochastically and generally follows the divergence of marker genes whereas gene gain (primarily, via HGT) is more episodic (Snel et al., 2002; Novichkov et al., 2004).

## Does evolution advance complexity?

The idea of a general evolutionary trend toward increasing complexity is extremely popular among both lay public and scientists and certainly was shared by Darwin who wrote, for example, in famous quote: “as natural selection works solely by and for the good of each being, all corporeal and mental endowments will tend to progress toward perfection” (Darwin, 1859). This view does not imply any mysterious strive for perfection as imagined by some pre-Darwinian biologists including Lamarck (1809) or teleology of any kind. Nevertheless, Darwin's position does suggest a trend of evolution from simple to complex forms which is indeed a highly intuitive notion that has some obvious support in well known facts of the history of life on earth. For example, the most organizationally complex organisms with the largest genomes, animals, and plants, appear only at relatively late stages of evolution. Even more generally, at the earliest stages in the evolution of life, origin of complex structures, such as the cell itself, “from so simple a beginning” (Darwin, 1859) appears inevitable. Thus, notwithstanding the numerous cases of reductive evolution, in particular among parasites and symbionts, the belief in a general complexification trend in the evolution of life appears to be common.

However, is complexification the prevailing modality of evolution? Phylogenomic reconstruction, at least for bacteria and Archaea, suggests otherwise. It is not surprising that differential gene loss dominates the evolution of commensal bacteria, such as Lactobacilli, from a complex free-living ancestor (Makarova et al., 2006). However, a qualitatively similar pattern was detected in evolutionary reconstructions for all bacteria and archaea (Snel et al., 2002; Mirkin et al., 2003; Makarova et al., 2007). Strikingly, more recent reconstructions that were performed using larger genome sets and more sophisticated computational methods confidently indicate that the genome of the last common ancestor of all extant archaea apparently was at least as large and complex as that of typical modern organisms in this domain of cellular life (Csuros and Miklos, 2009). Fully compatible reconstruction results have been reported for the expanded set of cyanobacterial genomes (Larsson et al., 2011). Thus, counter-intuitively, at least in prokaryotes, genome shrinkage that is sometimes called streamlining (Lynch, 2006) and is attributed to increasing selective pressure in successful, large populations (Lynch, 2006; Koonin, 2009b), appears to be is no less and probably more common than genome growth and complexification.

## The Wrightean-Darwinian-Lamarckian continuum of evolutionary processes

The Modern Synthesis of evolutionary biology emphasizes the randomness of mutations that provide the starting material for selection which engenders survival of the fittest under the given conditions and hence constitutes the adaptive, deterministic component of evolution. The insistence on such strict separation between the stochastic and deterministic aspects of evolution departs from Darwin's view that included the Lamarckian inheritance, with adaptive mutations directly caused by environmental cues, as an important, even if ancillary mechanism of evolution (Darwin, 1872).

Recently, several genetic phenomena with a distinct Lamarckian flavor have been discovered (Koonin and Wolf, 2009a; O'Malley and Koonin, 2011). Probably, the most striking case is the system of adaptive antivirus immunity, known as CRISPR-Cas (Clustered Regularly Interspaced Palindromic Repeats and CRISPR-associated proteins), that is present in most archaea and many bacteria (Koonin and Makarova, 2009; van der Oost et al., 2009; Marraffini and Sontheimer, 2010; Makarova et al., 2011). The CRISPR-Cas system integrates fragments of virus or plasmid DNA into a distinct, repetitive locus in the archaeal or bacterial genome. The transcript of this unique spacer functions as a guide RNA that is incorporated into a specific complex of Cas proteins possessing DNAse activity and directs this complex to the cognate alien DNA (or RNA) molecules that are cleaved and accordingly inactivated. The CRISPR-Cas system is amazingly efficient, with only about 10^−5^ failure rate (Deveau et al., 2008). This mechanism qualifies CRISPR-Cas as an adaptive immunity system, i.e., immunity system that adapts to a specific infectious agent, a novelty in prokaryotes (Koonin and Makarova, 2009; Bikard and Marraffini, 2012). Furthermore, the Lamarckian principle of inheritance and evolution is apparent in the mechanism of CRISPR-Cas function. Indeed, this system directly responds to an environmental cue (in this case, foreign DNA) by introducing a genetic change into the genome that is immediately adaptive with respect to that particular cue.

The discovery of the CRISPR-Cas immune system that functions on the Lamarckian principle drew attention to other phenomena that also seem to contain a Lamarckian component (Koonin and Wolf, 2009a; O'Malley and Koonin, 2011). Some of the common, central evolutionary processes such as HGT and stress-induced mutagenesis show a “quasi-Lamarckian” character. Indeed, even if HGT cannot be viewed as being directly caused by a specific environmental factor, it certainly is the case that the repertoire of the acquired genes depends on the environment. Genes common in a given environment will be acquired often and are likely to possess adaptive value. Stress-induced mutagenesis is triggered directly by environmental stress factors, e.g., desiccation or radiation, and produces variation that is required to develop resistant phenotype (Rosenberg and Hastings, 2003; Ponder et al., 2005; Galhardo et al., 2007; Galhardo and Rosenberg, 2009). The mutations are not specific to the biologically relevant loci but the activity of the molecular machineries of stress-induced mutagenesis [the best characterized of which is the SOS repair-mutagenesis system in bacteria (Sutton et al., 2000)] generates clusters of mutations, thus locally amplifying variability and so increasing the chance of adaptation once a single mutation appears in a relevant gene (Galhardo et al., 2007).

**Figure 6:**
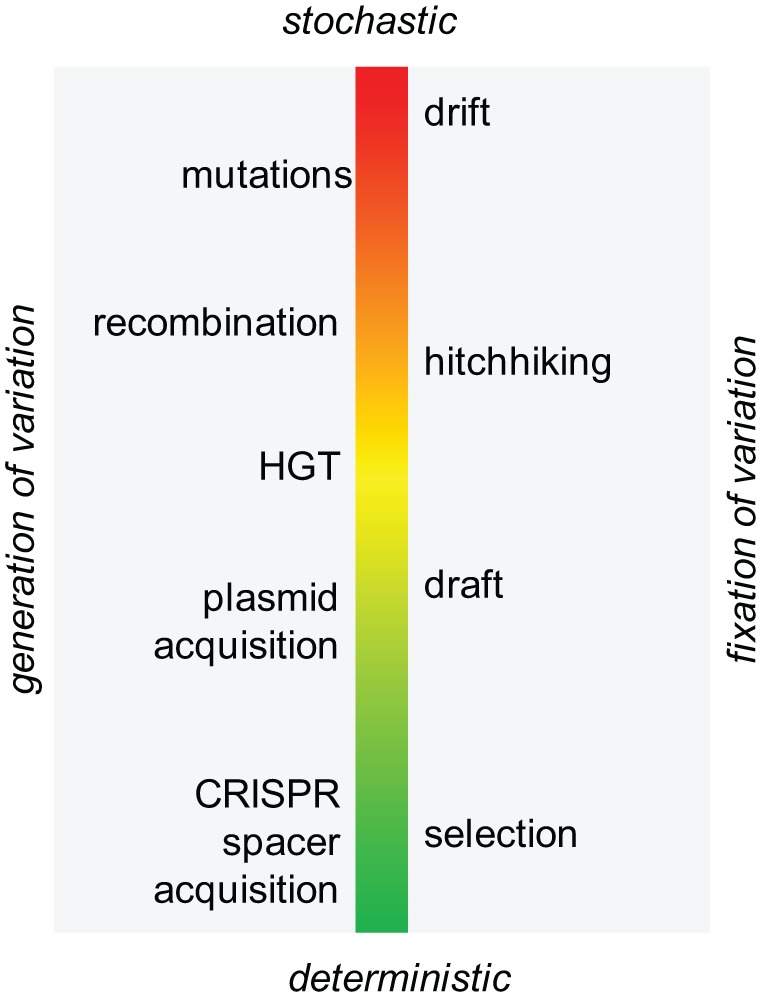
**The continuum of evolutionary processes, from stochasticity to determinism**.

More generally, recent empirical and theoretical studies of diverse processes of stochastic and deterministic change in genomes make it clear that evolution is not limited to the basic Darwinian scheme of random variation that is subject to selection. Evolution can be more adequately depicted as a continuum of processes from completely random ones, under the Wrightean modality defined by random variation and random fixation of changes via genetic drift; to the Darwinian modality with random changes fixed by the deterministic process of selection; to the Lamarckian mode in which both variation and fixation are deterministic (Figure 6) (Koonin and Wolf, 2009a; O'Malley and Koonin, 2011).

## Evolution of evolvability: dedicated mechanisms for evolution

All organisms possess a certain degree of evolvability, i.e., the ability to evolve. At the most basic level, evolvability stems from the theoretical impossibility of error-free replication. Genomic variation in evolving organisms is created by a combination of intrinsic replication errors, recombination and mutations induced external agents (mutagens). An intriguing, fundamental question in evolutionary biology is whether or not evolvability itself can evolve under selection, or put another way, whether there are dedicated mechanisms of evolution (Kirschner and Gerhart, 1998; Poole et al., 2003; Pigliucci, 2008; Brookfield, 2009). The prevailing wisdom among biologists seems to be that evolvability is not selectable but is simply maintained at a sufficient level by inevitable errors at all levels of biological information processing. Under this view, selection is always directed at minimization of the error rate but the ability to attain perfection is limited by genetic drift resulting in sufficient evolvability (Lynch, 2011). Evolutionary biologists are usually suspicious of the evolution of evolvability, generally under the old adage, “evolution has no forecast.”

Nevertheless, evidence in support of “evolvability of evolvability” is mounting. The very existence of complex molecular systems for stress-induced mutagenesis (error-prone repair) the activity of which is exquisitely regulated in response to stress implies that mechanisms enhancing variation when variation is needed for survival have evolved (Galhardo et al., 2007). Another remarkable mechanism that appears to have specifically evolved to generate variation involves the Diversity Generating Retroelements (DGR) (Medhekar and Miller, 2007). Strikingly, the DGR are found both in bacteriophages where they generate diversity in cell attachment surface proteins via reverse transcription-mediated mutagenesis, resulting in host tropism switching (Doulatov et al., 2004; Guo et al., 2008), and in bacteria themselves where they produce receptor variation leading to bacteriophage resistance (Bikard and Marraffini, 2012). The analogy between the activity of DGR and hypermutagenesis in animal immune systems is obvious except that the variation generated by the DGR is inherited.

Many bacteria and some archaea possess the natural transformation ability (that was used in the Avery experiment) that requires specialized, complex pumps (recently denoted transformosomes) that internalize DNA from the environment (Claverys et al., 2009; Johnsborg and Havarstein, 2009; Kruger and Stingl, 2011). The transformation machinery potentially could be viewed as a device that evolved under selective pressure to enhance HGT (Johnsborg and Havarstein, 2009). However, one could argue that the enhancement of HGT is only a side effect of the evolution of the transformation system, its actual raison d'etre being the utilization of DNA as a rich source of replication substrates (or simply food). This argument hardly can hold with regard to the type 4 secretion systems (T4SS) that specialize in secretion of DNA from bacterial cells (Hamilton et al., 2005; Hamilton and Dillard, 2006). The recently discovered Gene Transfer Agents (GTAs) are even more striking devices for DNA donation (Paul, 2008; McDaniel et al., 2010; Lang et al., 2012). The GTAs are a distinct type of defective bacteriophages that package in the capsid not the phage genome (which remains integrated in the host chromosome) but rather apparently random pieces of the host chromosome. The GTAs have been discovered in diverse bacteria and archaea and have been shown to infect and transfer their genetic content to a broad range of cohabitating prokaryotes (McDaniel et al., 2010). It does not seem conceivable that GTAs are anything but dedicated HGT vehicles. An additional notable aspect of T4SS and GTAs is that these devices mediate donation rather than consumption of DNA, i.e., apparently can directly benefit other microbes (recipients) rather than the donor. This seemingly altruistic behavior can be explained in terms of group selection whereby the object of selection is an ensemble of organisms that jointly benefit from adaptive mutations rather than a single organism. Group selection is a controversial subject in evolutionary biology (Maynard Smith, 1998; Borrello, 2005; Leigh et al., 2010) but the existence of dedicated devices for DNA donation appears to be a strong argument in its favor.

The discovery of T4SS and GTAs may be the most clear-cut pieces of evidence supporting evolution of evolvability just as the CRISPR-Cas system is the showcase for Lamarckian evolution. However, the case for the evolution of mechanisms for evolution seems to be much more general (O'Malley and Koonin, 2011). Population genetic theory holds that under a broad range of conditions a clonal population is generally doomed to collapse through the action of Muller's ratchet, the irreversible accumulation of deleterious mutations leading to gradual decline in fitness (Leigh et al., 2010; Bachtrog and Gordo, 2004). The effect of Muller's ratchet that has been directly demonstrated in controlled evolutionary experiments on RNA viruses (Chao, 1990; Duarte et al., 1992) and on bacteria (Andersson and Hughes, 1996). The principal way to escape Muller's ratchet is to enhance recombination via sex (in the form of meiotic crossing over in eukaryotes and in the form of conjugation in prokaryotes) or HGT. Just as sex is generally viewed as a mechanism that evolved to counteract the ratchet, HGT may be best understood as a more general variation-generating process that is supported by various evolved mechanisms. At the risk of being provocative, sex indeed can be legitimately regarded as a specialized form of HGT. Clearly, evolution maintains HGT within the optimal range rather than at the maximum possible level because the latter would eliminate genome stability and wreak havoc into selected high-fitness ensembles of genes (O'Malley and Koonin, 2011). Mechanisms that counter HGT also have evolved: these are the same that provide resistance against virus infection including CRISPR-Cas and restriction-modification (Marraffini and Sontheimer, 2008; Gardner and Olson, 2012).

At a different level, an apparent mechanism of evolution involves unusual, stable phenotype modifications that are widespread in bacteria and lead to coexistence of two distinct phenotypes in a clonal population, the so-called bistability regimes (Dubnau and Losick, 2006; Veening et al., 2008a; Piggot, 2010). For instance, under limited nutrient supply, *Bacillus subtilis* will form two subpopulations of which only the smaller one has the capacity to sporulate and thus yields the only survivors when the conditions become incompatible with cell growth and division (Veening et al., 2008a,b; Lopez et al., 2009). The coexistence is epigenetically inherited across many bacterial generations, hence this phenomenon has become known as bistability. In theoretical and experimental models bistability is rationalized as “bet hedging”: for organisms that live in often and unpredictably changing environments, it is beneficial to maintain a small subpopulation of likely survivors even when their fitness is comparatively low under normal conditions (Veening et al., 2008a; de Jong et al., 2011; Libby and Rainey, 2011; Rainey et al., 2011). The cost of maintaining this subpopulation is more than compensated by the benefit of survival under adverse conditions. Thus, the evolution of the regulatory circuitry that supports bistability appears to be not just a case of evolution of an evolutionary mechanism but more specifically evolution of a kin selection mechanism or evolution of altruism in bacteria. The evolution of kin selection demonstrated by bet hedging is paralleled by the mechanism of altruistic suicide that virus-infected bacteria and archaea commit using the toxin-antitoxin or abortive infection defense systems (Makarova et al., 2009; Van Melderen and Saavedra De Bast, 2009; Hayes and Van Melderen, 2011). In this case, by killing themselves early, before the virus has a chance to replicate, the microbes save their kin from infection. The reality of kin selection, just as that of group selection, is often hotly debated by evolutionary biologists (Nowak et al., 2010; Bourke, 2011; Ferriere and Michod, 2011; Strassmann et al., 2011) but the bistability/bet-hedging phenomena and altruistic suicide in bacteria and archaea seem to plainly demonstrate not only the existence but the evolvability of this form of selection.

In parallel with experimental studies, several theoretical models have been developed that characterize evolvability as a selectable trait in fluctuating environments (Earl and Deem, 2004; Jones et al., 2007; Draghi and Wagner, 2008). Thus, on the whole, and general theoretical doubts notwithstanding, evolution of evolvability appears to be an intrinsic and fundamental, if still poorly understood, aspect of the evolutionary process.

## The vast, ancient world of viruses

Viruses are no part of the modern synthesis or more generally the traditional narrative of evolutionary biology. Until very recently, viruses have been viewed primarily as pathogens of animals, plants, and bacteria. Several lines of recent discovery have radically changed this view and promoted viruses to a central position on the stage of evolution. This change in the evolutionary status of viruses and related selfish genetic elements has been discussed in detail elsewhere (Claverie, 2006; Koonin et al., 2006, 2011; Raoult and Forterre, 2008). Here we quickly recapitulate several key points, with a focus on the importance of viruses for evolutionary biology in general. Metagenomic and ecological genomics studies have shown that, astonishingly, viruses are the most common biological entities on earth (Edwards and Rohwer, 2005; Suttle, 2005, 2007). Viruses and/or virus-like mobile elements are present in all cellular life forms. Strikingly, in mammals sequences derived from mobile elements and endogenous viruses account for at least 50% of the genome whereas in plants this fraction can reach 90% (Feschotte et al., 2002; Kazazian et al., 2004; Devos et al., 2005; Hedges and Batzer, 2005). Even the genomes of some unicellular eukaryotes, such as *Trichomonas vaginalis*, consist mostly of inactivated transposons (Carlton et al., 2007; Pritham et al., 2007). Recruitment of mobile element sequences for transcription regulation and other cellular functions such as microRNA formation is a common phenomenon the full extent of which is not yet fully appreciated (Jordan et al., 2003; Piriyapongsa et al., 2007; Lisch and Bennetzen, 2011). Although genomes of prokaryotes are not so overwhelmed by mobile elements, due to the intense purifying selection, nearly all of them encompass multiple prophages and mobile elements. Notably, deletion of all prophages leads to a substantial drop of fitness in *E. coli* (Wang et al., 2010).

In at least some common environments such as ocean water and soil, the number of virus particles exceeds the number of cells by factors of 10–100 (Edwards and Rohwer, 2005; Suttle, 2007; Srinivasiah et al., 2008; Breitbart, 2012). Similarly, the genetic diversity of viruses, measured as the number of distinct genes, substantially exceeds the genetic diversity of cellular life forms. Furthermore, viruses, in particular bacteriophages, are major biogeochemical agents. Periodical killing of microbes, in particular cyanobacteria, has been identified as a major contributor to sediment formation and major contributors to the nutrient cycles in the biosphere (Suttle, 2007; Rohwer and Thurber, 2009). The same process obviously is a key determinant of the population dynamics of the hosts that shapes the selection-drift balance throughout the course of evolution (Weinbauer and Rassoulzadegan, 2004).

The very fact that viruses greatly outnumber bacteria in the environment implies that antivirus defense systems are central to the evolution of bacteria and archaea. This is indeed the case as made evident by the remarkable proliferation of diverse antivirus systems including CRISPR-Cas discussed above as well as multiple restriction-modification, abortive infection, toxin-antitoxin and other, still poorly characterized defense systems that in different combinations and with different abundances are present in most prokaryotes (Juhas et al., 2009; Labrie et al., 2010; Makarova et al., 2011; Martinez-Borra et al., 2012). Taken together, these findings and theoretical considerations strongly support the view that the virus-host arms race is one of the principal processes in all evolution (Forterre and Prangishvili, 2009; Stern and Sorek, 2011).

**Figure 7:**
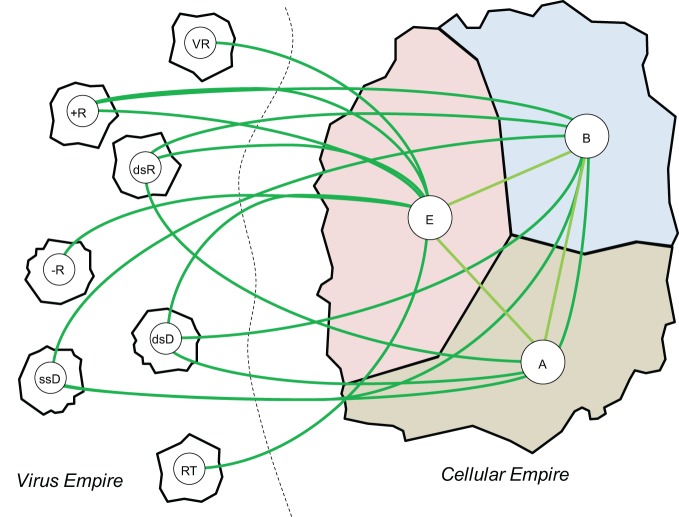
**The viral and cellular “empires” of life forms and domains within them.** The cellular empire domains: A, Archaea; B, Bacteria; E, Eukaryota. The Virus empire domains: +R, positive-strand RNA viruses; −R, negative-strand RNA viruses; dsR, double-stranded RNA viruses; dsD, double-stranded DNA viruses; ssD, single-stranded DNA viruses; RT, retro-transcribing elements/viruses; VR, viroids.

With regard to the classification of life forms, the only defensible position appears to be that viruses (and related mobile elements) and cells are the two principal categories of biological organization (Figure 7) (Raoult and Forterre, 2008; Koonin, 2010; O'Malley and Koonin, 2011); this view is independent of the semantic issue of viruses being “alive” or not (Koonin et al., 2009; Moreira and Lopez-Garcia, 2009; Raoult, 2009). These two categories of biological entities can be characterized as informational (genetic) parasites, i.e., viruses and other selfish elements, and genetically self-sustained organisms, i.e., cellular life forms. Mathematical modeling indicates that genetic parasites inevitably emerge in any replicator system (Szathmary and Maynard Smith, 1997; Takeuchi and Hogeweg, 2012). This conclusion is certainly intuitively plausible: one expects that cheaters will appear in any system with limited resources—in particular, in any system of replicators, such parasites will attempt to utilize the replication machinery without making it (Koonin and Martin, 2005). Also, the notion that virus-like selfish elements are an intrinsic part of life since its inception [which can be reasonably considered to coincide with the origin of replication (O'Malley and Koonin, 2011)] is compatible with the ubiquity of these elements in nature. In mathematical modeling, the outcome of the virus-host interaction depends on the specific parameters of the adapted model. In homogeneous models, virus-like parasites tend to cause collapse of the entire systems but in models with compartmentalization, which are most relevant for the actual evolution of life, stable host-parasite coexistence is possible (Takeuchi and Hogeweg, 2009). Moreover, the destructive effect of genetic parasites on the host is mitigated when a dedicated genetic information storage medium evolves, which could be one of the driving forces behind the evolution of DNA in the primordial RNA world (Takeuchi et al., 2011).

Further support for the classification of viruses as one of the two “empires” of life is the diversity of the replication-expression cycles that is found among viruses and related elements. Indeed, while cellular life forms all use a uniform replication-expression strategy based on double-stranded (ds)DNA replication, transcription of genes into mRNA or non-coding RNA, and translation of mRNA into protein, viral genome can be represented by all known forms of nucleic acids, and alternative replication processes such as RNA replication and reverse transcription are widely used (Figure 7) (Koonin et al., 2006). Finally, although viral genomes are generally small compared to the genomes of cellular life forms (viruses being the ultimate genetic parasites), the range of genomic complexity is remarkable, from only about 300 nucleotides and no genes in the simplest virus-like parasites, the viroids, to over a megabase and more than 1000 genes (genomes that are more complex than those of many bacterial parasites and symbionts) in the giant mimiviruses (Raoult et al., 2004; Colson et al., 2012). Overall, the conclusion is inescapable that the entire history of life is a story of perennial interplay between genetic parasites and their hosts that is a major driver of evolution for both biological empires.

## Evolution of microbes and viruses: a new evolutionary paradigm?

Prokaryotes (bacteria and archaea) and viruses entered the realm of evolution with the advent of genomics. Has the comparative study of these relatively simple (compared to eukaryotes) organisms radically changed the core tenets of evolutionary biology that were first envisaged by Darwin and were augmented with the genetic foundation in the Modern Synthesis? In terms of Kuhn's concept of the development of science (Kuhn, 1962), did the study of microbial evolution engender a paradigm shift?

It is not easy to answer this question definitively, possibly because the paradigm shift model does not adequately describe the evolution of biology (regardless of whether or not it fits the evolution of physics). Probably, a more appropriate epistemological framework is that of integration, i.e., a relatively smooth incorporation of the classic concepts into the new, more general and versatile theoretical constructs. This model of the evolution of science was recognized by Kuhn himself in his later work (Kuhn, 2002) and was recently examined by O'Malley in the context of biology (O'Malley, 2012; O'Malley and Soyer, 2012). The phylogenomic study of microbes and viruses uncovered new biological realms which Darwin and even the authors of the Modern Synthesis could not possibly fathom. The modes of evolution of these relatively simple organisms that, as we now realize, have dominated the biosphere since its beginning about 4 billion years ago to this day (and into any conceivable future) are different from the evolutionary regimes of animals and plants, the traditional objects of (evolutionary) biology. The study of microbial evolution has shattered the classic idea of a single, all-encompassing tree of life by demonstrating that the evolutionary histories of individual genes are generally different. Remarkably, however, these developments have not rendered trees irrelevant as a key metaphor of evolution (O'Malley and Koonin, 2011). Rather, they have shown that the bona fide unit of tree-like evolution is an individual gene not a genome, and a “tree of life” can only be conceived as a statistical trend in the “forest” of gene trees (Koonin and Wolf, 2009b). Tree-like evolution is a fundamental implication of the binary replication of the genetic material, so it served Darwin well to use a tree as the single illustration of his book. Without, obviously, knowing anything of DNA replication, Darwin grasped the central principle of the evolution of life, descent with modification, and the tree pattern followed naturally.

Microbiology yielded the first clear-cut case of Lamarckian evolution, the CRISPR-Cas system, and subsequent re-examination of other evolutionary phenomena (in both prokaryotes and eukaryotes) has strongly suggested that the (quasi)Lamarckian modality is common and important in all evolving organisms, completing the range of evolutionary phenomena from purely stochastic (drift, Wrightean evolution) to deterministic (Lamarckian evolution). Again, these findings not so much overturned but rather expanded the vision of Darwin who seriously considered Lamarckian mechanisms as being ancillary to natural selection (only the Modern synthesis banished Lamarck).

Crucially, the study of microbial evolution presented apparently undeniable cases of evolution of evolvability such as the GTAs and the DGRs. Moreover, the discovery of bet-hedging strategies and altruistic suicide in bacteria shows that kin selection (a subject of considerable controversy in evolutionary biology) is evolvable as well. Again, as in the case of Lamarckian mechanisms, these discoveries force one to re-examine many more phenomena and realize that evolution is not limited to fixation of random variation and survival of the fittest but rather is an active process with multiple feedback loops, and that dedicated mechanisms of evolution exist and themselves evolve. This is a major generalization that substantially adds to the overall structure of evolutionary biology but one has to realize that the principle of descent with modification remains at the core of all these complex evolutionary phenomena.

We now realize that evolution of life is to a large extent shaped by the interaction (arms race but also cooperation) between genetic parasites (viruses and other selfish elements) and their cellular hosts. Viruses and related elements, with their distinctive life strategy, informational parasitism, actually dominate the biosphere both physically and genetically, and represent one of the two principal forms of life that as intrinsic to the history of the biosphere as cells are. This new dimension of evolution simply could not be perceived by Darwin or even the creators of the Modern Synthesis due to the lack of relevant data.

**Figure 8:**
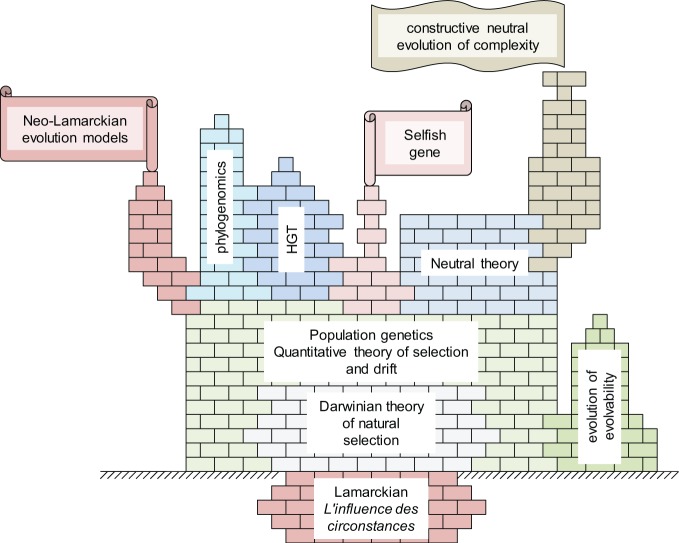
**The conceptual structure of evolutionary biology: the Darwinian core and the new levels of complexity**.

Thus, we are inclined to view the change in evolutionary biology brought about by phylogenomics of microbes and viruses as a case of integration rather than an abrupt departure from the paradigm of the Modern Synthesis (Figure 8). Darwin realized the importance of descent with modification and the tree pattern of evolution it implies whereas Fisher, Wright, and Haldane derived the laws of population genetics that still constitute the core of our understanding of evolution. However, recent advances, in particular those of microbial phylogenomics, added multiple, new and interconnected layers of complexity (Figure 8) such that the conceptual core is but a small part of the current big picture of evolutionary biology.

## Author contributions

Eugene V. Koonin and Yuri I. Wolf wrote the manuscript.

### Conflict of interest statement

The authors declare that the research was conducted in the absence of any commercial or financial relationships that could be construed as a potential conflict of interest.
